# Impact of Bivalirudin on Ischemia/Reperfusion Injury in Patients with Reperfused STEMI Assessed by Cardiac Magnetic Resonance

**DOI:** 10.3390/ph17020196

**Published:** 2024-02-01

**Authors:** Yizhi Zhang, Zhiguo Zou, Bihe Xu, Binghua Chen, Heng Ge, Song Ding, Jun Pu

**Affiliations:** 1 Department of Cardiology, Shanghai Renji Hospital, Shanghai Jiaotong University School of Medicine, Shanghai 200002, China; zyzzfklily@sjtu.edu.cn (Y.Z.); zouzhiguo@renji.com (Z.Z.); 54xbh@163.com (B.X.); dr.geheng@foxmail.com (H.G.); 2 Department of Radiology, Shanghai Renji Hospital, Shanghai Jiaotong University School of Medicine, Shanghai 200002, China; chenbinghua@renji.com; 3 Department of Cardiology, Punan Branch of Renji Hospital, Shanghai Jiaotong University School of Medicine, Shanghai 200127, China

**Keywords:** bivalirudin, STEMI, ischemia/reperfusion injury, cardiovascular magnetic resonance

## Abstract

Thrombin is an important ischemia/reperfusion injury (IRI) mediator in patients with ST-elevation myocardial infarction (STEMI). This study examines the use of bivalirudin, a direct thrombin inhibitor, in reducing IRI in STEMI patients. STEMI patients (*n* = 21) were treated with bivalirudin and compared to 21 patients treated with unfractionated heparin (UFH) from the EARLY Assessment of Myocardial Tissue Characteristics by CMR in STEMI (EARLY-MYO-CMR) registry (NCT03768453). Infarct size (IS) and left ventricular ejection fraction (LVEF) were comparable between the two groups at follow up. During the first cardiac magnetic resonance (CMR) scan within the first week after percutaneous coronary intervention (PCI), all patients in both the bivalirudin and UFH groups exhibited myocardial edema. However, the myocardium edema volume was significantly less in the bivalirudin group (*p* < 0.05). At the one-month follow-up, a smaller proportion of patients in the bivalirudin group than in the UFH group exhibited myocardial edema (4.7% vs. 33.3%, *p* < 0.05). At the three-month follow-up, myocardial edema had completely resolved in the bivalirudin group, while it persisted in two patients in the UFH group. The incidence and volume of microvascular obstruction (MVO) were significantly lower in the bivalirudin group during the acute phase. Additionally, the incidence of intramyocardial hemorrhage (IMH) was significantly lower in the bivalirudin group during both the acute and follow up (*p* < 0.05). These findings were corroborated by T2 and T1 mapping results. The study concluded that the use of bivalirudin for anticoagulation is associated with attenuated IRI in STEMI patients who receive primary PCI.

## 1. Introduction

The primary goal of ST-segment elevation myocardial infarction (STEMI) management is to reduce the risk of death and the extent of permanent cardiac injury associated with MI. Current recommendations call for STEMI patients to receive timely percutaneous coronary intervention (PCI). Anticoagulant therapy is an essential component in the implementation of primary PCI for patients presenting with STEMI. Current guidelines endorse the use of unfractionated heparin (UFH) as anticoagulants in patients undergoing PCI with a Class IC recommendation [1].

However, the perioperative administration of UFH has been associated with an increased risk of bleeding and is contraindicated in patients with heparin-induced thrombocytopenia (HIT). Consequently, several alternatives have been developed in recent years to mitigate these risks. Bivalirudin, a novel direct thrombin inhibitor, has been explored as a potential substitute for UFH in patients with STEMI undergoing primary PCI, especially those at a heightened risk of bleeding or diagnosed with HIT (Class IC) [2].

Empirical research has shown that the use of bivalirudin during PCI not only mitigates the risk of bleeding complications but also correlates with a decrease in all-cause mortality, a reduction in reinfarction rates, and an enhancement in cardiac function post-revascularization, compared to UFH [3,4]. However, due to the variability in the dosage and duration of bivalirudin administration across these studies, it remains a matter of debate whether bivalirudin can offer additional clinical benefits beyond a reduced risk of bleeding.

The MATRIX trial indicated that a strategy of bivalirudin monotherapy, compared to UFH with or without glycoprotein IIb/IIIa inhibitors, did not result in a decrease in major adverse cardiovascular events or net adverse clinical events in patients with acute myocardial infarction (AMI) [5]. However, a subsequent analysis of five clinical trials and the HORIZONS-AMI trial suggested that extending the administration of bivalirudin at a full-PCI dose post-PCI was associated with the lowest risk of ischemic events and improved cardiac function following revascularization, compared to UFH [4,6]. The most recent randomized clinical trial, BRIGHT-4, revealed that anticoagulation with bivalirudin, followed by a high-dose infusion of bivalirudin for 2–4 h post-PCI, significantly reduced the 30-day composite rate of all-cause mortality compared to heparin monotherapy [3]. Based on these pieces of evidence, the European Society of Cardiology upgraded bivalirudin’s recommendation for routine usage in STEMI treatment to Class IIa [1]. The pathophysiological mechanisms contributing to these benefits beyond hemorrhage reduction, however, remain elusive.

Prompt reperfusion is the paramount strategy for the preservation of ischemic myocardium. Nevertheless, reperfusion itself instigates myocardial ischemia/reperfusion injury (IRI), typified by an acute inflammatory response orchestrated by activated inflammatory cells, chemokines, cytokines, and adhesion molecules [7]. The extent and severity of IRI are pivotal determinants of long-term prognosis post-STEMI.

A multitude of studies have substantiated that thrombin, irrespective of its procoagulant properties, mediates IRI in the endothelium and cardiomyocytes in various organs including the lung, cerebrum, and heart [8,9,10]. It has been reported that during the onset of STEMI, there is a significant escalation in thrombin generation, which is further augmented during the reperfusion process. This heightened thrombin generation post-STEMI is correlated with a subsequent unfavorable prognosis post-reperfusion [11]. As a result, anti-thrombin therapy has emerged as a potential therapeutic target against IRI following primary PCI. Pan et al. reported that sustained thrombin inhibition in cardiac IRI maintains vascular integrity, mitigates the extent of hemorrhage, and prevents the no-reflow phenomenon [12].

Unlike UFH which achieves anticoagulation through its interaction with antithrombin III (AT-III), bivalirudin exerts its effect by directly inhibiting thrombin. This is achieved through its specific binding to both the catalytic site and anion-binding exosite of circulating and clot-bound thrombin [13]. In vitro studies have demonstrated that lepirudin, an analog of bivalirudin, can prevent cell death induced by thrombin in a dose-dependent manner [14]. Furthermore, recombinant hirudin, another direct thrombin inhibitor, has been observed to enhance myocardial recovery during the immediate postischemic period [15]. Our previous research indicated that in a mouse model, bivalirudin could block thrombin-induced endothelial hyperpermeability, an effect not observed with UFH [16]. However, these effects have not been evaluated in STEMI patients who received bivalirudin during primary PCI. We postulated that the clinical advantages previously associated with bivalirudin are due to its direct antithrombin effect, which may subsequently preserve endothelial integrity from thrombin following reperfusion, a benefit not provided by UFH. As such, the purpose of this study is to scrutinize the protective role of bivalirudin in IRI among STEMI patients who have undergone immediate reperfusion.

## 2. Results

### 2.1. Baseline Clinical Characteristics

After propensity score-matching (PSM), patients who received bilirubin and UFH shared comparable demographic and clinical variables (Table 1). Both groups were similar in terms of age, gender, health conditions, and PCI-associated treatment details. Most patients were given the P2Y12R inhibitor Ticagrelor, and all patients received aspirin for anti-platelet activity. No GPIIb/IIIa inhibitor was used. All subjects had a thrombolysis in myocardial infarction (TIMI) flow grade of 3 post-PCI.

### 2.2. Quantitative and Qualitative Features of IRI in Acute Phase and Follow-Up

**Figure 1:**
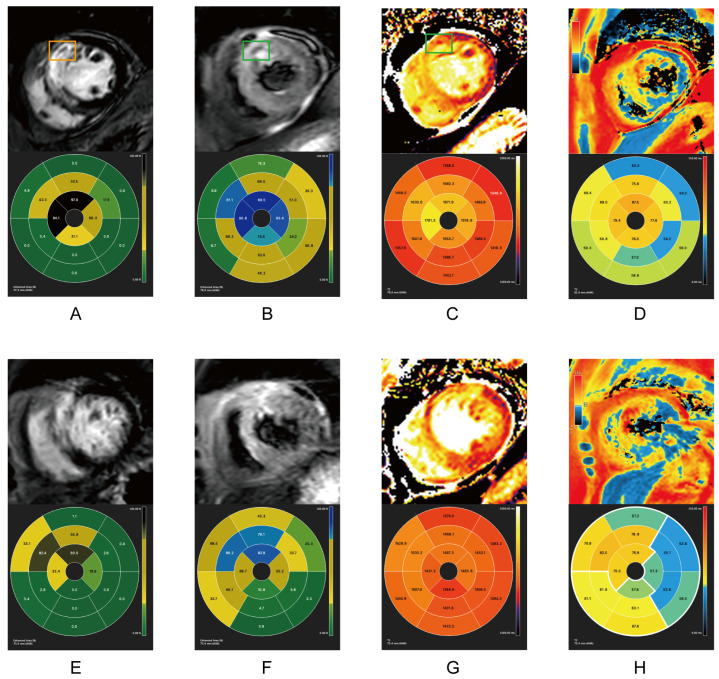
CMR indexes within one week after PCI in two groups. Top row: UFH group. Bottom row: Bivalirudin group. (**A**,**E**) LGE; (**B**,**F**) T2-STIR; (**C**,**G**) T1 mapping; (**D**,**H**) T2*. Orange rectangle in A: MVO identified in the LGE sequence. Green rectangle in (**B**,**C**): IMH identified in T2-STIR and T1-mapping sequence. The signals from CMR images were depicted as corresponding proportions in LGE and T2-STIR, and as absolute time in T1 mapping and T2*, all of which were based on the 17-segment model (AHA). CMR: cardiovascular magnetic resonance. PCI: Percutaneous intervention. UFH: Unfractionated heparin. LGE: Late gadolinium enhancement. IMH: Intramyocardial hemorrhage. T2-STIR: T2-weighted short-tau triple inversion-recovery. AHA: American Heart Association.

**Figure 2:**
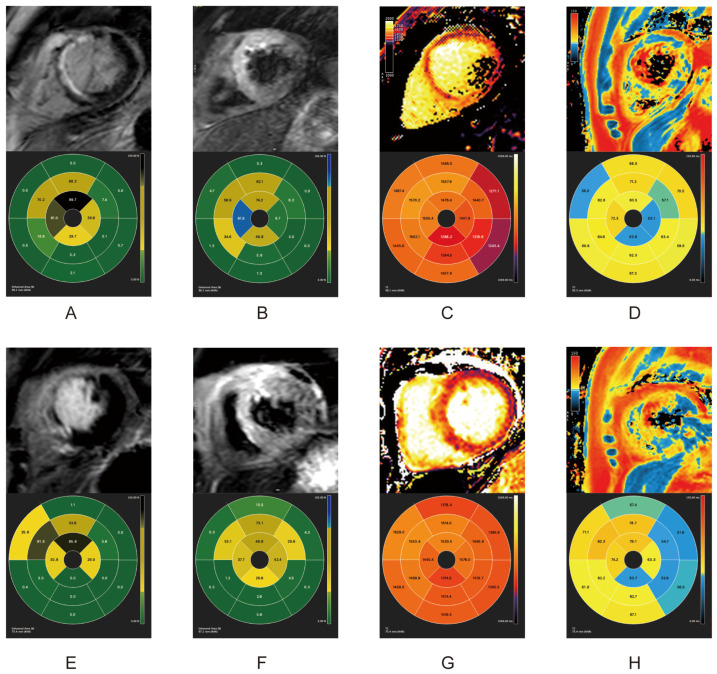
CMR indexes one month after PCI in two groups. Top row: UFH group. Bottom row: Bivalirudin group. (**A**,**E**) LGE; (**B**,**F**) T2-STIR; (**C**,**G**) T1 mapping; (**D**,**H**) T2*. Orange rectangle in A: MVO identified in the LGE sequence. Green rectangle in (**B**,**C**): IMH identified in T2-STIR and T1-mapping sequence. The signals from CMR images were depicted as corresponding proportions in LGE and T2-STIR, and as absolute time in T1 mapping and T2*, all of which were based on the 17-segment model (AHA). CMR: cardiovascular magnetic resonance. PCI: Percutaneous intervention. UFH: Unfractionated heparin. LGE: Late gadolinium enhancement. IMH: Intramyocardial hemorrhage. T2-STIR: T2-weighted short-tau triple inversion-recovery. AHA: American Heart Association.

**Figure 3:**
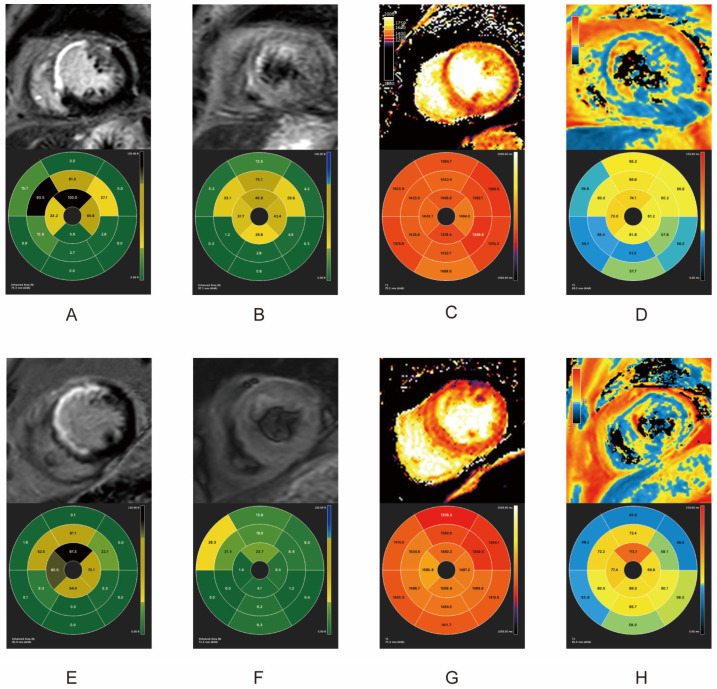
CMR indexes three months after PCI in two groups. Top row: UFH group. Bottom row: Bivalirudin group. (**A**,**E**) LGE; (**B**,**F**) T2-STIR; (**C**,**G**) T1 mapping; (**D**,**H**) T2*. Orange rectangle in A: MVO identified in the LGE sequence. Green rectangle in (**B**,**C**): IMH identified in T2-STIR and T1-mapping sequence. The signals from CMR images were depicted as corresponding proportions in LGE and T2-STIR, and as absolute time in T1 mapping and T2*, all of which were based on the 17-segment model (AHA). CMR: cardiovascular magnetic resonance. PCI: Percutaneous intervention. UFH: Unfractionated heparin. LGE: Late gadolinium enhancement. IMH: Intramyocardial hemorrhage. T2-STIR: T2-weighted short-tau triple inversion-recovery. AHA: American Heart Association.

All 21 patients had cardiovascular magnetic resonance (CMR) scans within one week (acute phase, average scan time 5.8 days) and one and three months after primary PCI. As shown in Table 2, during the acute and follow-up phases, both groups shared similar infarct sizes (IS) and left ventricular ejection fraction (LVEF) levels. All patients in both groups appeared to have myocardial edema within one week after reperfusion. Patients undergoing PCI with bivalirudin use presented with significantly lower incidences (Fisher’s exact test, *p* < 0.05), and levels of myocardial edema (Student’s *t* test, *p* = 0.02), MVO, and IMH (Fisher’s exact test, *p* < 0.05) than patients using UFH. One month after PCI, only one patient in the bivalirudin group still had myocardial edema on the T2-weighted sequence, while in the UFH group, seven patients still presented myocardial edema. MVO was absent in both groups at one month. At the three-month follow-up, none of the patients in the bivalirudin group presented with myocardial edema, while two patients in the UFH group still had myocardial edema in the T2-weighted sequence. The IMH results were consistent in both the acute phase and one month after PCI. The lower incidence of IMH in the bivalirudin group was consistent at all time points, regardless of whether IMH was identified by T2-STIR or T2* (Table 2, Fisher’s exact test, all *p* < 0.05). CMR indexes in the acute and chronic phases in of two patients in the two groups are illustrated in Figure 1, Figure 2 and Figure 3.

Compared with the UFH group, bivalirudin usage was associated with a shorter overall T2 time and a longer infarct core T1 time in the acute phase and at one month (*p* < 0.05). There were no T1 and T2 mapping differences between the groups at the three-month follow-up (Figure 1 and Table 3). The results of T2 mapping indicated a higher level of myocardial edema in the UFH group in the acute phase and at the one-month follow-up. The shorter infarct core T1 time in the UFH group might be a consequence of the higher incidence of MVO because the region of MVO usually presents a shortened T1 time [17]. (Figure 1 and Table 3).

Within one week after the revascularization, IS measured by LGE showed comparable results (Figure 1A,E). The selected patient in the UFH group had MVO (orange rectangle). Despite sharing similar IS, the patients receiving bivalirudin had much less myocardial edema, as measured by T2-STIR (Figure 1B,F), T1 mapping (Figure 1C,G), and T2 mapping (Figure 1D,H). Remarkably, the patients in the UFH group also had IMH (Figure 1B–D, green rectangle). Approximately one month post-PCI, IS in both groups remained comparable. The MVO in patients in the UFH group was fully replaced by scar tissue in the LGE sequence (Figure 2A,E). The patients who received bivalirudin had a much lower level of myocardial edema at one month post PCI as measured by T2-weighted sequencing (Figure 2B,F), T1 mapping (Figure 2C,G), and T2 mapping (Figure 2D,H). Three months later, IS in both groups remained comparable (Figure 3A,E). The patients who received bivalirudin only had a minimal volume of myocardial edema (Figure 3B), while the patients who received UFH still had residual myocardial edema even three months after PCI (Figure 3F), which was also reflected in T1 mapping (Figure 3C,D) and T2 mapping (Figure 3D,H).

## 3. Discussion

Primary PCI is the standard-of-care in STEMI patients. UFH and bivalirudin are the two most widely used procedural anticoagulants during primary PCI. Prior studies have predominantly concentrated on the safety profiles of these two anticoagulant regimens. To our understanding, this is the first research exploring the differences in impact on IRI between these two anticoagulants. Our novel findings are that compared with UFH, the perioperative administration of bivalirudin resulted in a significantly reduced degree of myocardial edema, MVO, and IMH, both acutely and at one to three months after reperfusion. However, it did not influence IS or LVEF during the subsequent observation period. Our research suggests a potential preference for the utilization of bivalirudin as an anticoagulant during PCI for STEMI, when juxtaposed with UFH.

Thrombin has been identified as a potential mediator of IRI in the myocardium, with its generation significantly increased following STEMI [10]. Previous studies have indicated that cardiomyocyte death, associated with thrombin, can be mitigated through the use of thrombin inhibitors such as lepirudin, hirudin, and antithrombin nanoparticles [12,14,18]. Anti-thrombin approaches also have IRI protective effects in other organs. For instance, Vargas et al. demonstrated that inhibition of thrombin by Proline-Phenylalanine-Arginine-Chloromethyl-Ketone (PPACK) through nanoparticles can help preserve the tissue microarchitecture and function after the onset of acute kidney injury or STEMI [19,20]. Namachivayam et al. also showed that targeted inhibition of thrombin by a nanomedicine-based approach was protective without increasing interstitial hemorrhages in the inflamed bowel or other organs [21].

Our prior experimental data have underscored the protective influence of bivalirudin against thrombin-induced endothelial hyperpermeability [16]. In the present study, we observed that patients with STEMI who received bivalirudin during primary PCI, as compared to their PSM counterparts who were administered UFH, exhibited reduced myocardial edema, MVO, and IMH during both acute and chronic phases. This study underscores the benefits of perioperative bivalirudin administration on IRI and proposes a potential pathological mechanism that may explain the clinical advantages of bivalirudin in reperfused STEMI patients.

In the realm of IRI, it has been conclusively demonstrated that the degree of myocardial edema is directly correlated with adverse cardiovascular outcomes in patients with STEMI [22,23]. In the present study, we observed a significantly reduced area of edema in the bivalirudin group compared to the UFH group, as delineated by T2-STIR sequences, both in the acute phase and at the one-month follow-up. This reduction in myocardial edema was further corroborated by shortened T2 mapping within the bivalirudin cohort.

Previous research has established that a key mechanism of IRI-induced edema is the increase in endothelial permeability [24]. During ischemic events, the integrity of interendothelial cell junctions is maintained. However, reperfusion disrupts these junctions, leading to an increase in vascular permeability [25]. The phenomenon of thrombin-induced hyperpermeability, first observed several decades ago, has been identified as a significant factor contributing to myocardial edema in IRI. Itagaki et al. demonstrated that the use of a sphingosine kinase inhibitor in coculture could mitigate the endothelial permeability induced by thrombin [26]. Our prior research has shown that thrombin can induce an increase in sphingosine 1-phosphate (S1P) and S1P receptor 2 (S1PR2), both of which play crucial roles in endothelial hyperpermeability. Bivalirudin, by inhibiting S1PR2 expression, can reverse thrombin-induced permeability [16]. Hirudin, another direct thrombin inhibitor, has also been reported to prevent vascular endothelial cell apoptosis and permeability [27]. These findings may provide some insight into the potential molecular mechanisms underlying the reduced myocardial edema observed in the bivalirudin group compared with the UFH group in this study.

MVO, often termed as “no reflow”, is a common occurrence in reperfused AMI [28]. It was initially identified as a reperfusion phenomenon. MVO, as defined by CMR, aligns well with angiographic and pathological evidences [29,30]. It is a significant prognostic indicator in STEMI patients, marking adverse prognosis [31,32]. In our study, we observed a typical pattern of contrast-enhanced infarct area with a contrast-void infarct core, termed MVO. We discovered that the MVO area within one week post-primary PCI was notably smaller in the bivalirudin group compared to the UFH group. This could be attributed to less severe myocardial edema in the bivalirudin group, a known causal factor of MVO [33].

IMH, a common complication post-reperfusion in STEMI, is characterized by erythrocyte leakage and is associated with adverse outcomes. IMH leads to iron deposition in the myocardium, negatively impacting long-term prognosis. These iron degradation products can be detected as areas with shortened T2* time in chronicphase CMR [34]. In the acute phase, IMH is traditionally recognized as a hypointense area surrounded by a hyper-intensive area in T2-weighted sequences. Although evidence has emerged showing inconsistency among CMR approaches to identifying IMH, in this study, T2-STIR in the acute phase and T2* in the chronic phases yielded identical results [35]. This may be explained by the limited sample size.

In this research, the bivalirudin group exhibited a significantly lower incidence of IMH post-primary PCI, with only one case (4.8%) within a month, compared to the UFH group’s 33.3%. Additionally, the shortened T2* time at the one-month and three-month follow-ups after reperfusion in the UFH group implied that more iron content was detected, which may reflect worse myocardial injury compared with that in the bivalirudin group. It is generally believed that IMH is a downstream phenomenon of MVO since MVO almost always precedes IMH [23,36]. IMH could be the consequence of severe MVO by capillary destruction and extravasation of red blood cells into the myocardium. In this study, the lower IMH incidence in the bivalirudin group was consistent with findings of lower MVO incidence and volume compared with the UFH group. Bivalirudin yielded a lower incidence of IMH coinciding with decreased edema at the acute phase and one-month follow-up, which could herald an improved outcome after STEMI. The reduction in IMH could be attributed to bivalirudin’s direct thrombin inhibition, considering the role of thrombin-induced microvascular damage in IMH pathogenesis [37]. Our findings were consistent with other observations. It has been reported that continued thrombin inhibition by PPACK, a potent and irreversible direct thrombin inhibitor, can maintain endothelial integrity, thereby mitigating vascular leakage, hemorrhage, and no-reflow phenomena [12]. These pieces of evidence suggest that the protective effects of bivalirudin may be due to its direct thrombin inhibition, a property not exhibited by UFH.

Studies have shown that MVO are associated with adverse LV remodeling in reperfused STEMI patients [37]. IMH, a severe MVO outcome, has also been significantly linked with a decrease in LVEF in STEMI patients post-PCI [36]. Notably, MVO and IMH have been identified as stronger independent predictors of infarct zone contractile recovery than IS or transmural extent [38]. In our research, despite bivalirudin usage correlating with reduced myocardial edema and lower MVO and IMH incidence, no significant LVEF difference was observed between the two groups during follow-up, potentially due to the limited sample size and short follow-up period. Hence, a blind study with a larger cohort and longer follow-up time is still warranted.

The limitations of this study are primarily attributed to the constrained sample size, a consequence of the retrospective observational nature of the study. Within the confines of the EARLY-MYO-CMR registry (NCT03768453), a mere 21 patients were administered bivalirudin as an anti-coagulant and received CMR scan within one week, one month, and three months after PCI. To substantiate these findings, it is imperative that future research undertakes a comprehensive, head-to-head randomized clinical trial with a significantly larger sample size. This will provide a more robust and reliable understanding of the subject matter.

To summarize, we found that peri-PCI usage of bivalirudin was associated with less myocardial IRI than that of UFH.

## 4. Materials and Methods

### 4.1. Study Design and Subjects

The data utilized in this study were procured from the EARLY Assessment of Myocardial Tissue Characteristics by CMR in STEMI (EARLY-MYO-CMR) registry (NCT03768453). This registry is a multicenter, prospective database that encompasses all patients diagnosed with STEMI who have undergone CMR examination.

The study’s inclusion criteria were as follows: patients experiencing their first STEMI episode, characterized by typical ischemic syndromes and electrocardiography manifestation of ST elevation in at least two contiguous precordial leads (≥2 mm) or peripheral leads (≥1 mm); successful primary PCI within 12 h of symptom onset; and CMR scans conducted within the first week and at one and three months post-reperfusion. All patients adhered to standard medical treatment in accordance with current guidelines.

The study subjects were patients who received bivalirudin as an anticoagulant during primary PCI. Propensity score-matched (PSM) patients from the registry who received UFH during primary PCI as an anticoagulation were matched based on the covariates delineated in Table 1. The propensity score was estimated using logistic regression. We used one-to-one nearest-neighbor matching without replacement and a caliper width of 0.2 of the standard deviation of the logit of the propensity score. This rigorous selection process aimed to control for confounding variables and mimic randomization in this retrospective observational study.

### 4.2. Usage of Bivalirudin and UFH

Following an initial dose of aspirin and either 180 mg of ticagrelor or 300 mg of clopidogrel, patients underwent immediate coronary angiography. During PCI, either bivalirudin (1.75 mg/kg bolus and subsequent 1.75 mg/kg/h for the procedure’s duration) or UFH (1000 U/kg bolus with an additional 1000 U per hour) was administered. Thrombus aspiration was not routinely conducted, and only culprit vessels were addressed during PCI. GPIIb/IIIa inhibitors were used selectively at the discretion of the attending physician.

### 4.3. CMR Protocols

Cardiovascular magnetic resonance (CMR) serves as a dependable, noninvasive method for assessing IRI in the myocardium following reperfusion [39]. This study leverages the capabilities of CMR to gauge the extent and intensity of IRI. The parameters measured include the infarct size (IS), the presence of myocardial edema, intramyocardial hemorrhage (IMH), microvascular obstruction (MVO), and the left ventricular ejection fraction (LVEF). These measurements provide a comprehensive evaluation of IRI, underscoring the pivotal role of CMR in this context.

CMR-based parameters were determined as surrogate IRI markers according to the latest expert consensus [40]. All CMR images were obtained using a 3.0 Tesla magnetic resonance scanner (Achieva TX; Philips Healthcare, Amsterdam, The Netherlands). All images were analyzed offline using commercially available software (CVI42; Circle Cardiovascular Imaging, Inc., Calgary, AB, Canada) by two experienced cardiologists blinded to the study design. The exact protocols we used to obtain each image aligned well with other articles published by our research team [41,42].

The cine images for LVEF measurement, including 16 slices in short axis, that were used to calculated LVEF were acquired by steady-state free precession (SSFP) sequence using flip angle 45°, repetition time (TR) 2.8 ms, echo time (TE) 1.4 ms, field of view (FOV) 300 × 300 mm, voxel size 1.2 × 1.2 × 7 mm^3^, and gap 0. LVEF was obtained using short axis cine images with open contours included in both systolic and diastolic phase. The consistency of the myocardium weight results in both phases and by both analyzers (±5 g) was used to measure the quality and accuracy of the LVEF results.

The LGE dataset was acquired following intravenous injection of gadobutrol (0.1 mmol/kg, Gadavist, Bayer Healthcare Pharmaceuticals Inc., Leverkusen, Germany). A phase-sensitive inversion recovery (PSIR) sequence was subsequently undertaken at 10 min. The slice thickness was 8 mm with a 2 mm gap. The inversion times were delicately selected to ensure adequate nulling of normal myocardium (characteristically between 280 mm and 400 mm). The IS was derived from LGE as the volume of myocardium with a signal intensity > 5*SDs higher than the mean signal intensity in the remote myocardium. MVO was defined as a hypointense core inside an area of hypointense LGE and was manually delineated on short-axis slices.

Myocardial edema was measured using a dark-blood T2-weighted short-tau triple inversion-recovery (T2-STIR), where edema was recognized as an area with signal intensity 2*SDs higher than that of remote myocardium (TE 90 ms, TR two R-R intervals, flip angle 90°, acquired spatial resolution 1.37 × 1.37 × 10 mm^3^). The presence of IMH was defined on T2-STIR as a hypointense core within the edema myocardium in the acute phase and shortened regressed T2* time on the T2* sequence in the one and three month follow-up CMR scan.

### 4.4. Statistical Analysis

For the analysis of human data, continuous variables were tested for normality by using a combination of graphical methods, including histograms, Q–Q plots, and the Shapiro–Wilk test. Normally distributed variables are expressed as the mean ± SD, and variables with skewed distribution are expressed as the median with the corresponding IQR. Categorical variables are displayed as frequencies (percentages). Differences in continuous data were compared by using Student’s *t* test or the Mann–Whitney U test, and categorical variables were compared by using the chi-squared test or Fisher’s exact test when appropriate. Because the use of thrombin inhibitors was determined by the physician’s decision and not randomized, a propensity score-matched (PSM) analysis was further used to match the baseline and clinical characteristics of the two groups.

Statistical analyses were conducted in R version 4.2.0 (R Foundation for Statistical Computing, Vienna, Austria). A *p* value < 0.05 (2-sided) was considered statistically significant.

## 5. Conclusions

Our research demonstrated that the peri-procedural administration of bivalirudin, as opposed to UFH, was linked to a reduction in myocardial IRI during both the acute and follow-up post-reperfusion in patients with STEMI. This was observed despite no significant differences in IS and LVEF between the bivalirudin and UFH groups. The implications of our findings suggest a potential preference for bivalirudin over UFH during PCI in STEMI patients. Nevertheless, a larger, double-blind clinical trial with a longer follow-up duration is necessary to validate these conclusions.

## Figures and Tables

**Table 1:** Demographic and clinical information of the included patients.

Indexes	All (*n* = 42)	Bivalirudin Group (*n* = 21)	UFH Group (*n* = 21)	*p* Value
Age, years (SD)	67.9 (5.5)	67.7 (6.5)	68.1 (4.1)	0.762
Male, *n* (%)	32 (76.2)	16 (76.2)	16 (76.2)	1.000
Smoking, *n* (%)	25 (59.5)	13 (61.9)	12 (57.1)	0.753
Hypertension, *n* (%)	28 (66.7)	14 (66.7)	14 (66.7)	1.000
Hyperlipidemia, *n* (%)	24 (57.1)	12 (57.1)	12 (57.1)	1.000
Diabetes, *n* (%)	12 (28.6)	6 (28.6)	6 (28.6)	1.000
BMI, kg/m^2^ (SD)	25.3 (1.6)	25.1 (1.7)	25.6 (1.6)	0.366
Killip class, *n* (%)				0.948
I	35 (83.3)	18 (86.7)	17 (81.0)	
II	3 (7.1)	1 (4.8)	2 (9.5)	
III	2 (4.8)	1 (4.8)	1 (4.8)	
IV	2 (4.8)	1 (4.8)	1 (4.8)	
Location of infarction, *n* (%)				1.000
Anterior	26 (61.9)	13 (61.9)	13 (61.9)	
Non-anterior	16 (38.1)	8 (38.1)	8 (38.1)	
Multivessel disease, *n* (%)	21 (50.0)	11 (52.4)	10 (47.6)	0.758
Number of lesions, *n* (%)				1.000
1	26 (61.9)	13 (61.9)	13 (61.9)	
≥2	16 (38.1)	8 (38.1)	8 (38.1)	
Thrombus aspiration, *n* (%)	10 (23.8)	6 (28.6)	4 (19.0)	0.469
TIMI flow post PCI, *n* (%)				-
0	0 (0)	0 (0)	0 (0)	
1	0 (0)	0 (0)	0 (0)	
2	0 (0)	0 (0)	0 (0)	
3	42 (100)	21 (100)	21 (100)	
Aspirin, *n* (%)	42 (100)	21 (100)	21 (100)	1.00
P2Y12R inhibitor, *n* (%)				0.378
Clopidogrel	6 (14.3)	2 (9.5)	4 (19.0)	
Ticagrelor	36 (85.7)	19 (90.5)	17 (81.0)	
GPIIb/IIIa inhibitor usage	0 (0)	0 (0)	0 (0)	-

SD: standard deviation; BMI: body mass index.

**Table 2:** CMR index comparisons during the follow-up period.

CMR Index	All (*n* = 42)	Bivalirudin Group (*n* = 21)	UFH Group (*n* = 21)	*p* Value
Within one week after PCI				
LVEF, % (SD)	44.4 (7.0)	44.0 (6.9)	44.9 (6.8)	0.67
IS, % (SD)	29.6 (5.9)	28.4 (5.9)	30.9 (5.7)	0.17
Myocardium edema, *n* (%)	42 (100)	21 (100)	21 (100)	-
Myocardium edema, % (SD)	34.2 (7.4)	31.5 (7.4)	36.9 (6.9)	0.02
MVO, *n* (%)	26 (61.9)	12 (57.1)	14 (66.7)	0.75
MVO, % (SD)	0.9 (0.5)	0.5 (0.4)	1.2 (0.3)	<0.01
IMH *, *n* (%)	8 (19.0)	1 (4.8)	7 (33.3)	0.02
T2 mapping, ms (SD)	48.8 (7.5)	46.11 (3.2)	51.5 (9.3)	0.02
T1 mapping				
Infarct core, ms (SD)	1247.9 (96.1)	1284.0 (49.8)	1211.8 (115.6)	0.01
Peri-infarct zone, ms (SD)	1319.1 (107.4)	1296.1 (110.8)	1342.0 (98.7)	0.17
Remote myocardium, ms (SD)	1339.1 (147.2)	1306.8 (119.9)	1371.9 (163.7)	0.16
One month after PCI				
LVEF, % (SD)	48.2 (10.6)	49.2 (11.6)	47.3 (9.3)	0.57
dLVEF, % (SD)	3.8 (6.6)	4.3 (6.8)	3.3 (6.3)	0.63
IS, % (SD)	23.4 (6.4)	22.7 (5.8)	24.1 (6.8)	0.50
Myocardium edema, *n* (%)	8 (19.0)	1 (4.8)	7 (33.3)	0.02
Myocardium edema, % (SD)	9.7 (3.7)	5.4 (-)	10.3 (3.6)	-
MVO, *n* (%)	0 (0)	0 (0)	0 (0)	-
MVO, % (SD)	0 (0)	0 (0)	0 (0)	-
IMH *, *n* (%)	8 (19.0)	1 (4.8)	7 (33.3)	0.02
T2 mapping, ms (SD)	43.9 (8.5)	40.4 (5.2)	47.4 (9.6)	<0.01
T1 mapping				
Infarct core, ms (SD)	1242.6 (93.9)	1274.9 (53.7)	1210.2 (112.5)	0.02
Peri-infarct zone, ms (SD)	1309.0 (113.7)	1286.8 (115.1)	1331.2 (107.2)	0.21
Remote myocardium, ms (SD)	1330.4 (151.5)	1298.7 (123.9)	1362.1 (168.9)	0.18
Three months after PCI				
LVEF, % (SD)	49.5 (11.1)	50.1 (11.8)	48.9 (10.3)	0.72
dLVEF, % (SD)	5.1 (7.6)	5.3 (6.9)	5.0 (8.1)	0.89
IS, % (SD)	23.2 (8.7)	22.7 (6.6)	23.7 (10.4)	0.71
Myocardium edema, *n* (%)	2 (4.8)	0 (0)	2 (9.5)	-
Myocardium edema, % (SD)	1 (2.4)	0 (0)	1 (4.76)	-
MVO, *n* (%)	0 (0)	0 (0)	0 (0)	-
MVO, % (SD)	0 (0)	0 (0)	0 (0)	-
IMH *, *n* (%)	8 (19.0)	1 (4.8)	7 (33.3)	0.02
T2 mapping, ms (SD)	42.6 (9.6)	40.1 (8.6)	45.1 (9.8)	0.09
T1 mapping				
Infarct core, ms (SD)	1287.0 (96.1)	1306.0 (56.0)	1267.9 (121.0)	0.21
Peri-infarct zone, ms (SD)	1288.2 (107.3)	1282.7 (118.5)	1293.7 (94.5)	0.75
Remote myocardium, ms (SD)	1306.3 (156.0)	1270.9 (131.6)	1341.8 (169.8)	0.15

UFH: Unfractionated heparin; PCI: Percutaneous intervention; SD: standard deviation; CMR: Cardiovascular magnetic resonance; LVEF: Left ventricular ejection fraction; dLVEF: ΔLeft ventricular ejection fraction; IS: Infarct size; MVO: Microvascular obstruction; IMH: Intramyocardial hemorrhage; Ms: Milliseconds. * IMH was measured by T2-STIR within one week and T2* one and three-months after PCI.

**Table 3:** T1/T2 mapping comparison of the included patients.

T1/T2 Mapping	All (*n* = 42)	Bivalirudin Group (*n* = 21)	UFH Group (*n* = 21)	*p* Value
Within one week after PCI				
T2 mapping, ms (SD)	48.8 (7.5)	46.11 (3.2)	51.5 (9.3)	0.02
T1 mapping				
Infarct core, ms (SD)	1247.9 (96.1)	1284.0 (49.8)	1211.8 (115.6)	0.01
Peri-infarct zone, ms (SD)	1319.1 (107.4)	1296.1 (110.8)	1342.0 (98.7)	0.17
Remote myocardium, ms (SD)	1339.1 (147.2)	1306.8 (119.9)	1371.9 (163.7)	0.16
One month after PCI				
T2 mapping, ms (SD)	48.8 (7.5)	46.11 (3.2)	51.5 (9.3)	0.02
T1 mapping				
Infarct core, ms (SD)	1247.9 (96.1)	1284.0 (49.8)	1211.8 (115.6)	0.01
Peri-infarct zone, ms (SD)	1319.1 (107.4)	1296.1 (110.8)	1342.0 (98.7)	0.17
Remote myocardium, ms (SD)	1339.1 (147.2)	1306.8 (119.9)	1371.9 (163.7)	0.16
Thirty days after PCI				
T2 mapping, ms (SD)	42.6 (9.6)	40.1 (8.6)	45.1 (9.8)	0.09
T1 mapping, ms (SD)				
Infarct core, ms (SD)	1287.0 (96.1)	1306.0 (56.0)	1267.9 (121.0)	0.21
Peri-infarct zone, ms (SD)	1288.2 (107.3)	1282.7 (118.5)	1293.7 (94.5)	0.75
Remote myocardium, ms (SD)	1306.3 (156.0)	1270.9 (131.6)	1341.8 (169.8)	0.15

SD: standard deviation; UFH: Unfractionated heparin; Ms: Milliseconds.

## Data Availability

Data is contained within the article.
